# A Simple Scope‐Advancement Technique to Correct Pancreatic–Biliary Duct Axis Misalignment During Pancreatic Guidewire‐Assisted Biliary Cannulation

**DOI:** 10.1002/ccr3.73155

**Published:** 2026-07-13

**Authors:** Yuichi Takano, Naoki Tamai, Jun Noda, Tetsushi Azami, Fumitaka Niiya, Masatsugu Nagahama

**Affiliations:** ^1^ Division of Gastroenterology, Department of Internal Medicine Showa Medical University Fujigaoka Hospital Yokohama Kanagawa Japan

**Keywords:** biliary cannulation, ductal axis misalignment, ERCP, pancreatic guidewire‐assisted cannulation

## Abstract

Recognition of pancreatic–biliary duct axis misalignment is important when pancreatic guidewire‐assisted biliary cannulation is unsuccessful. A simple scope‐advancement maneuver under fluoroscopic guidance can redirect the catheter toward the bile duct axis and facilitate biliary access without additional devices, potentially avoiding the need for more advanced rescue techniques.

## 1 Case Presentation

An 83‐year‐old man with distal bile duct stenosis underwent ERCP (Figure 1). Pancreatic guidewire (PGW)‐assisted biliary cannulation failed because the catheter remained aligned with the pancreatic duct axis (Figure 2). Advancing the scope deeper into the duodenum with slight upward and rightward angulation redirected the catheter toward the bile duct axis under fluoroscopic guidance, enabling successful biliary cannulation (Figures 3 and 4, Video 1).

**FIGURE 1:**
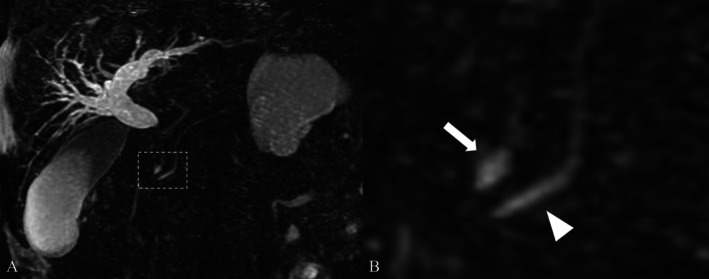
(A) Magnetic resonance cholangiopancreatography demonstrated a stricture in the middle bile duct, with a short segment of normal distal bile duct remaining between the stricture and the papilla. (B) Magnified view of the dotted square in panel A, demonstrating the axis of the common bile duct (arrow) and the main pancreatic duct (arrowhead).

**FIGURE 2:**
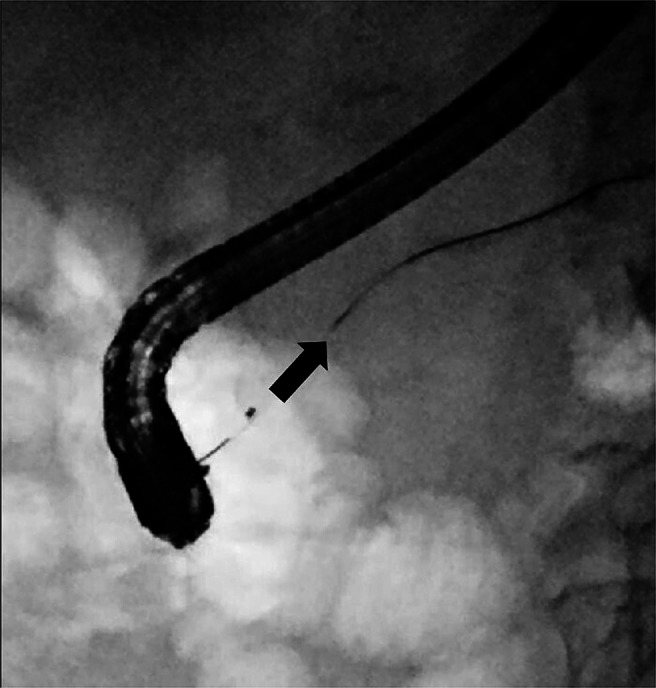
Fluoroscopic image demonstrating that the catheter is aligned with the pancreatic duct axis (arrow), preventing biliary access.

**FIGURE 3:**
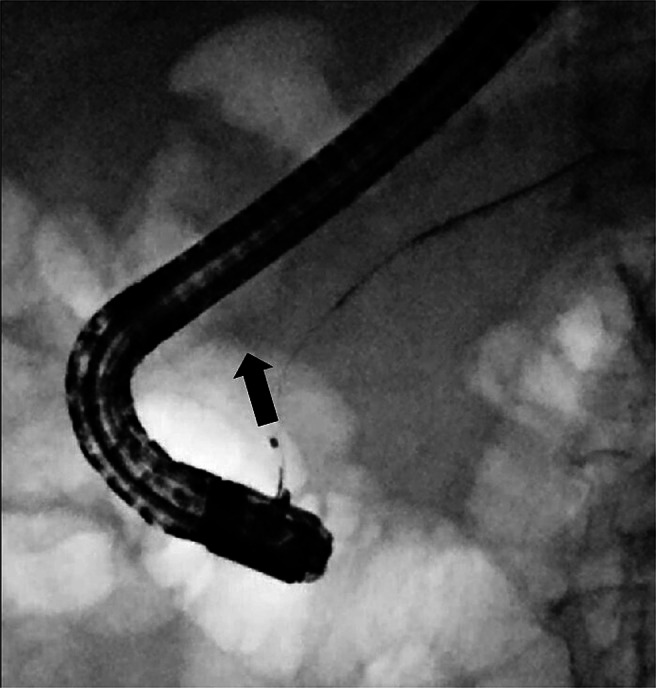
After scope advancement, the catheter orientation shifts toward the bile duct axis under fluoroscopic guidance (arrow).

**FIGURE 4:**
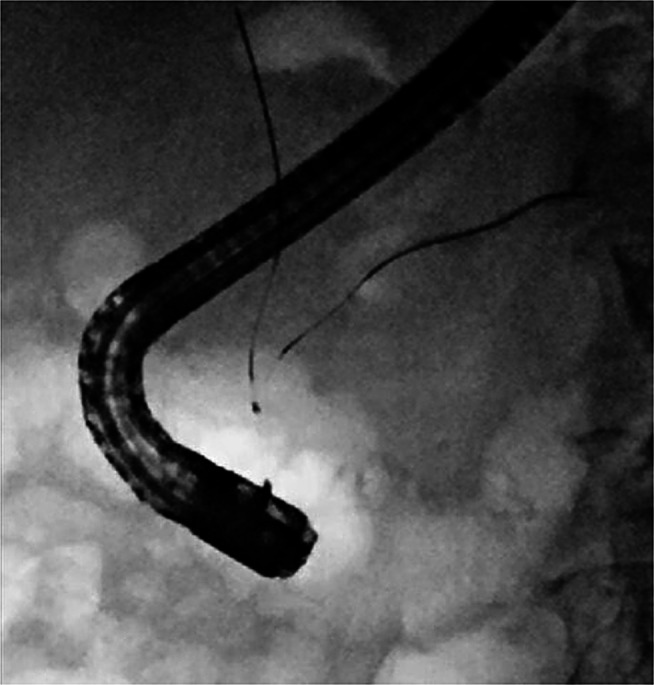
Successful deep biliary cannulation.

**VIDEO 1:** A simple scope‐advancement technique to correct pancreatic–biliary duct axis misalignment during pancreatic guidewire‐assisted cannulation. Video content can be viewed at https://onlinelibrary.wiley.com/doi/10.1002/ccr3.73155.

Although advanced rescue techniques such as precut sphincterotomy are effective for difficult biliary cannulation, they require greater expertise and may increase the risk of post‐ERCP pancreatitis [1, 2]. Recognition of pancreatic–biliary duct axis misalignment and simple scope‐position adjustment may facilitate biliary access before proceeding to more advanced interventions.

## Author Contributions

**Yuichi Takano:** conceptualization, writing – original draft, writing – review and editing, investigation. **Tetsushi Azami:** writing – review and editing. **Naoki Tamai:** writing – review and editing. **Jun Noda:** writing – review and editing. **Fumitaka Niiya:** writing – review and editing. **Masatsugu Nagahama:** writing – review and editing.

## Funding

The authors have nothing to report.

## Ethics Statement

The authors have nothing to report.

## Consent

Written informed consent was obtained from the patient to publish this report in accordance with the journal's patient consent policy.

## Conflicts of Interest

The authors declare no conflicts of interest.

## Data Availability

The data that support the findings of this study are available from the corresponding author upon reasonable request.
